# Graphene-multiferroic interfaces for spintronics applications

**DOI:** 10.1038/srep31346

**Published:** 2016-08-23

**Authors:** Zeila Zanolli

**Affiliations:** 1 Forschungszentrum Jülich, Peter Grünberg Institute (PGI-1) and Institute for Advanced Simulation (IAS-1), Jülich, D-52425, Germany; 2 Institute for Theoretical Solid State Physics, RWTH Aachen University and European Theoretical Spectroscopy Facility (ETSF), D-52056 Aachen, Germany

## Abstract

Graphene and magnetoelectric multiferroics are promising materials for spintronic devices with high performance and low energy consumption. A very long spin diffusion length and high carrier mobility make graphene attractive for spintronics. The coupling between ferroelectricity and magnetism, which characterises magnetoelectrics, opens the way towards unique device architectures. In this work, we combine the features of both materials by investigating the interface between graphene and BaMnO_3_, a magnetoelectric multiferroic. We show that electron charge is transferred across the interface and magnetization is induced in the graphene sheet due to the strong interaction between C and Mn. Depending on the relative orientation of graphene and BaMnO_3_, a quasi-half-metal or a magnetic semiconductor can be obtained. A remarkably large proximity induced spin splitting of the Dirac cones (~300 meV) is achieved. We also show how doping with acceptors can make the high-mobility region of the electronic bands experimentally accessible. This suggests a series of possible applications in spintronics (e.g. spin filters, spin injectors) for hybrid organic-multiferroic materials and reveals hybrid organic-multiferroics as a new class of materials that may exhibit exotic phenomena such as the quantum anomalous Hall effect and a Rashba spin-orbit induced topological gap.

Spintronics^1^ is the new paradigm of information technology. It aims at the construction of devices based on the spin degree of freedom of the electron instead of, or in addition to, the electron charge. Spintronics is a field in which huge potential for both innovative applications and fundamental research coexist and spur on each other’s development. In the spin FET design^2^, the possibility of manipulating the spin of electrons *via* an external electric field is proportional to the Spin Orbit Coupling (SOC) of the channel material. Conventional III–V semiconductors were originally proposed as channel materials due to their high SOC and long spin lifetime. However, since the mean velocity of the carriers is quite small, the corresponding spin diffusion length is relatively short and results in weak magnetoresistance signals^3^. Carbon-based nanomaterials, instead, are especially promising for use as channel materials in spintronic devices^4^. The low atomic number of Carbon entails small SOC and hyperfine interaction which, combined with high Fermi velocity, result in remarkably long (up to ~100 *μ*m) spin diffusion lengths^5^,^6^. Furthermore, the peculiar electronic and transport properties of C nanomaterials^7^,^8^ lead to high electron mobility and gate-tunable carrier concentration. On the other hand, small SOC limits the possibility of manipulating electrons *via* an external applied field. In addition, to achieve graphene-based transistors one needs to open its vanishing electronic gap. Isolated pristine graphene is not optimal for spintronics applications: it is necessary to combine it with a material with high SOC, and either cut it into nanoribbons^9^ or place it on a suitable substrate to open an electronic band gap^10^. This latter option can have several advantages. First, graphene can be grown or mechanically transferred onto substrates, which is easier than fabricating nanoribbons. For instance, epitaxial growth of oxides on graphene has been achieved for EuO^11^ and Mn_3_O_4_^12^. Second, the weak SOC of graphene can be enhanced and a spin polarization can be induced in graphene by interaction with a ferromagnetic oxide substrate (*proximity interaction*)^13^,^14^. Third, a substrate or a strong perpendicular electric field break the graphene inversion symmetry and lead to Rashba Spin-Orbit coupling^15^. Finally, gap tunability can be achieved by varying the perpendicular electric field, as has been shown for graphene on hexagonal BN^16^. The choice of a *magnetoelectric* (ME) multiferroic material as substrate can satisfy all these points. ME materials are characterised by a coupling between ferroelectricity and magnetization, hence allowing one to control the magnetization *via* an electric field^17^. This feature makes ME materials appealing for use in spintronic applications^18^.

This paper reports on the first principles investigation of the electronic and magnetic properties of the interface between graphene and ME multiferroics. We focus on manganite-based MEs, since the Mn *d* electrons carry a strong spin polarization. First-principles calculations have shown that Mn adatoms interact strongly with carbon *π* orbitals inducing Rashba SOC and quantum anomalous Hall effect in graphene^19^. Hexagonal 2H-BaMnO_3_ has been chosen as representative of this class of materials since it is ME^20^,^21^ and its experimental lattice constant matches that of graphene. First principles calculations^21^ show that the mechanism for improper ferroelectricity in 2H-BaMnO_3_ is the same as for the class of RMnO_3_ hexagonal manganites (where R is a rare earth element). We find that the graphene–BaMnO_3_ (g–BMO) hybrid system is spin polarized with magnetization and charge transfer induced in the carbon atoms due to the strong interaction with the surface Mn atoms. The most stable reconstruction is quasi-half-metallic and acts as an injector of 100% spin polarized carriers. The electronic properties at the Fermi energy (E_F_) are governed by the details of the interface. The graphene Dirac cones for majority and minority carriers present a huge splitting of about 300 meV. Furthermore, we show that both the energy at which the band crossing occurs and the splitting of spin-up/spin-down bands can be tuned by doping of the graphene monolayer.

Our first-principles calculations describe well the insulating nature and the magnetic properties of bulk BaMnO_3_, which is crucial in order not to short-circuit the graphene layer, ensure that electron transport will only occur through graphene and correctly model the spin polarization induced on the C network (see Supplementary Information, Table S2 and Figure S2). The magnetic moments of the Mn atoms are antiferromagnetically (AFM) coupled between planes in the [0001] direction and are oriented at 120° with respect to each other in the plane^20^. Here, a ferromagnetic collinear arrangement of the spins in the (0001) plane is assumed, as this is considered a good approximation to the experimental spin structure^21^,^22^. The computed magnetic moment on Mn atoms is 2.43 *μ*_*B*_. The ferroelectric ground state of 2H-BaMnO_3_ is also correctly reproduced, with a macroscopic polarization of 0.267 *μ*C/cm^2^ and 19.037 *μ*C/cm^2^ for relaxed and strained-to-graphene cases, respectively.

The Mn-terminated (0001) surface of BaMnO_3_ is highly reactive because each Mn atom has a +4 oxidation state. This surface will interact strongly with graphene, inducing transfer of charge and magnetization to the carbon network. Proximity-induced magnetization of graphene due to Mn adatoms has been predicted in ref. 19. A symmetric BaMnO_3_ slab cut in the plane orthogonal to the [001] direction (see Supplementary Information for non-trivial electrostatics) was relaxed starting from the bulk coordinates imposing a lattice constant 4 times that of graphene. To avoid spurious interaction between the two opposite surfaces we modeled a slab consisting of 13 atomic layers (Fig. 1a). The slab relaxed in a centrosymmetric structure, losing the initial ferroelectric polarization. The resulting system is a magnetic semiconductor (cfr. band structure in Fig. 1b), characterised by a very different electronic structure for the two spin channels. Majority spins present a direct gap at the Γ point (0.493 eV), while minority spins have an indirect gap between the K and M points (1.627 eV). Spin polarization of the slab is due surface Mn. Each of these atoms has two excess electrons that are donated to the oxygen in the bulk. This results in a large increase of the magnetic moment on surface Mn (4.29 *μ*_*B*_) with respect to the middle of the slab (2.41 *μ*_*B*_, close to the bulk value 2.43 *μ*_*B*_). The electrons at the surface give rise to the spin-polarized bands below E_F_ (Fig. 1b). A slight spin polarization also extends to the underlying BaO_3_ layers, which are non-magnetic in the bulk (Table 1).

Having clarified the electronic and magnetic properties of BaMnO_3_, we can now address those of its interface with graphene. The g–BMO slab is constructed by placing a 4 × 4 graphene supercell on either side of the strained BMO slab. Atomic relaxations with different graphene offsets lead to the most stable structure shown in Fig. 2a. Every Mn surface atom lies approximately in the middle of a Carbon-Carbon bond, at a vertical distance of about 1.84 Å. Such a short distance and the high binding energy of graphene (−274 meV per C) are a first indication of a strong covalent bond, which is confirmed by the analysis of the electronic band structure in terms of the orbital-projected bands (*fat bands*).

The g–BMO electronic band structure (Fig. 2d) is spin polarized and shows a quasi-half-metal character: minority spins have no gap, while majority spins present a 139 meV gap slightly above the Fermi level at the Γ point. The computed group velocity of the majority spins tends to zero near the gap, while the minority velocity reaches ~2.3 × 10^5 ^m/s for the band crossing E_F_: only minority carriers can propagate at the interface. The majority band edge lies slightly above E_F_ (78 meV), such that by applying a small gating potential, an injector of 100% spin polarised carriers is achieved (*spin filter*). The band contribution coming from each atom and orbital has been identified with projected bands. This technique (Supplementary Figure S3) demonstrates that bands in the [−1.25 eV, 0.25 eV] range are exclusively due to the graphene/BaMnO_3_ interface. In particular, a strong hybridisation is found among the C *sp*^2^, Mn 3*d* and O 2*p* states. Ba atoms do not contribute in a range of about 2 eV around E_F_, meaning that the results presented in this work are expected to hold for other hexagonal RMnO_3_ compounds.

As a consequence of this strong interfacial interaction, the surface Mn atoms induce spin polarization and charge transfer in graphene. The hybrid system is magnetic. As for the bare BaMnO_3_ slab, this is due to the excess electron charge carried by the surface Mn atoms, which makes their spin polarization higher (2.73 *μ*_*B*_) than that in the centre of the slab (2.42 *μ*_*B*_, similar to bulk), as shown in Fig. 3b and Table 1. An important difference with respect to the bare slab is that part of this excess charge is now transferred to graphene, inducing a spin polarization in carbon. The details depend on the specific position of each C atom with respect to the underlying substrate. Six carbon sublattices can be identified, as illustrated in Fig. 3d. All the carbon atoms belonging to a given sublattice have the same magnetic moment and charge transfer (Table 2). It is important to note that spin polarization is forced in “pristine” graphene: no defects, no strain, and no impurities are present. The effect is exclusively due to the interaction with the Mn-terminated surface and hence it can be achieved using other RMnO_3_ compounds as substrate. The magnetic moment on individual C atoms is at most 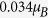, and the crucial result is that the electronic bands that contribute to electron transport in g–BMO (i.e. the bands in the vicinity of 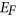) are strongly spin polarized (Fig. 2d). These results, obtained with LDA, are robust against the inclusion of the U correction on Mn 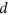 electrons, as detailed in the SI.

An important feature of the electronic structure is that the graphene Dirac cones are found at the K and K’ points of the 4 × 4 supercell. The latter are mostly contributed by C and, to a minor extent, by Mn and O. There is a remarkably large splitting of ~290 meV between spin up and down Dirac points. However, these high mobility regions occur at energies which are difficult to reach in experiments. Doping graphene with acceptors can shift E_F_ and, hence, the position of the Dirac cones. Several structures with one and two B substitutional dopants per graphene layer have been relaxed and analysed. The most likely site for a single dopant (3% *p*-type doping concentration) is sublattice “3” (Fig. 2b). When a second B is introduced (6% concentration) the most stable configuration occurs for B in sublattices “3” and “2” (Fig. 2c). Inspection of the electronic structure (Fig. 2d–f) shows that substitutional doping with acceptors achieves the desired effect of shifting the Dirac cones towards E_F_. In addition, the dopants break the symmetry of the graphene lattice and, hence, open a gap at the Dirac point of ~50–70 meV. In Yang^23^ magnetization was induced by proximity interaction with EuO and Dirac cones were at about −1.3 eV. However, the graphene-EuO system is always metallic and the shift of the Dirac cones towards *E*_F_ is only achieved when graphene is far from the substrate. A further advantage of doping is that the carrier velocity near K increases with doping for both spin channels: the velocity in g–BMO (3.5 × 10^5 ^m/s for majority, 5.3 × 10^5 ^m/s for minority) is close to pristine graphene (8.5 × 10^5 ^m/s). Velocities up to 4.2 × 10^5 ^m/s (majority) and 6.6 × 10^5 ^m/s (minority) are reached for 2 Boron dopants.

Other possible graphene/BaMnO_3_ interfaces have been checked. A metastable configuration is reached when one Mn lies in the middle of a C hexagon and two Mn are vertically aligned with C in the unit cell. This system is a magnetic semiconductor (Supplementary Figure S4) with a gap of 324 meV and 205 meV for majority and minority carriers, respectively. The Dirac cone splitting is still present and amounts to 200 meV.

Since the electronic band structures exhibit quite different features for majority and minority spins, very different electronic transport properties are expected as well. A possible device exploiting these results is a spin–FET in which spin injection is obtained by graphene-BaMnO_3_ in its ground state. Then, the FET channel can be made by graphene on an insulating material, such as hBN, which preserves the long intrinsic spin coherence length of carbon. A high SOC substrate would enable control of the spin in the channel. The continuous graphene with a modulated substrate minimizes problems with contact resistances and interface mismatch in the transport direction. Last but not least, the proximity coupling of graphene with the Mn-terminated surface of BaMnO_3_ may present a quantum anomalous Hall (QAH) effect and Rashba spin orbit splitting, analogously to graphene doped with Mn adatoms^19^. Calculations of the Berry curvature of the bands near the Dirac points could be used to determine the presence of a QAH state.

In conclusion, first-principles calculations have been used to investigate the interfaces between graphene and a magnetoelectric multiferroic material, BaMnO_3_. The strong interaction between graphene *π* electrons and the magnetic Mn *d* states induces a significant spin polarization in graphene. This interaction breaks the carbon network in six sublattices with different magnetic and charge transfer properties. The ground-state structure is a quasi-half-metal that allows for injection of 100% spin polarised carriers. Depending on the relative orientation of graphene and substrate, a magnetic semiconducting metastable state is also found. In each case, the graphene Dirac cones present a remarkably large spin splitting (300 meV). This high-mobility region (similar to suspended graphene) can be made accessible to experiment *via* substitutional doping of graphene with acceptors. The main features of the electronic structure do not depend on the Ba atom, and are expected to hold also for other hexagonal manganites RMnO_3_ or any magnetic insulator.

## Methods

All calculations have been performed using the SIESTA implementation^24^ of Density Functional Theory within the Local Spin Density Approximation (LSDA) using the Perdew and Zunger form^25^ of the Ceperley-Alder exchange-correlation functional^26^. Convergence (see Supplementary Information) of electronic structure, magnetism and polarization in bulk BaMnO_3_ is achieved for a real space grid cutoff of 1200 Ry, a shifted 6 × 6 × 12 k-point sampling of the Brillouin zone and a Fermi-Dirac smearing of 100 K in a 30-atom cell which is primitive for the ground state phase (space group *P*6_3_*cm*).

## Additional Information

**How to cite this article**: Zanolli, Z. Graphene-multiferroic interfaces for spintronics applications. *Sci. Rep*. **6**, 31346; doi: 10.1038/srep31346 (2016).

## Supplementary Material

Supplementary Information

## Figures and Tables

**Figure 1:**
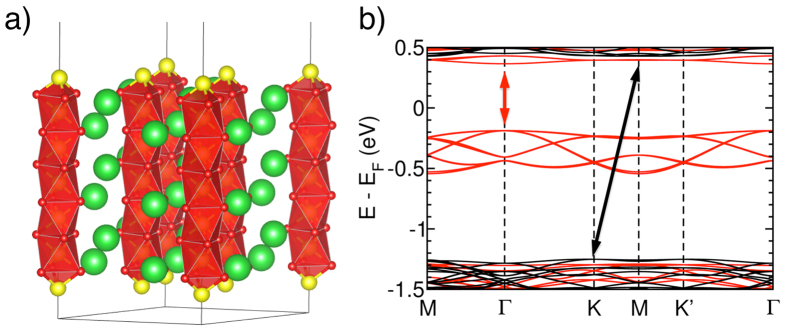
Structural and electronic properties of the BaMnO_3_ slab. (**a**) Side view of the relaxed BaMnO_3_ slab (color code: Mn yellow, Ba green, O red) and (**b**) its electronic band structure for majority (red) and minority (black) spins. The arrows indicate the band-gap for each spin polarization. The states below E_F_ are Mn surface states.

**Figure 2:**
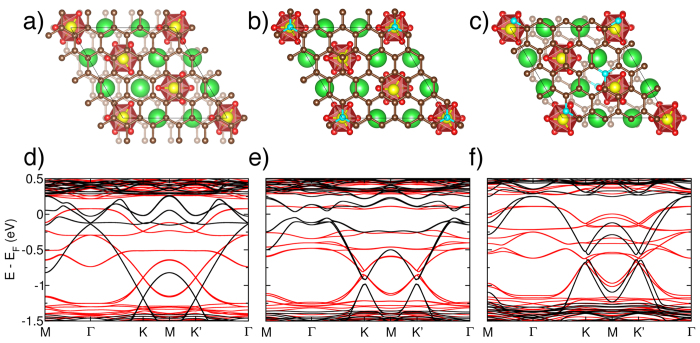
Structural and electronic properties of the graphene–BaMnO_3_ slab. Ball-and-stick model (top) and electronic band structure (bottom) of graphene–BaMnO_3_ in its ground state (left), and of the relaxed structure with one (middle) and two (right) substitutional Boron atoms. The position of the Dirac cones is shifted towards E_F_ with increasing doping. Red and black solid lines indicate, respectively, majority and minority spin channels. The Fermi energy of the hybrid system is taken as a reference. Atom colors: Mn yellow, Ba green, O red, C gold, B light blue.

**Figure 3:**
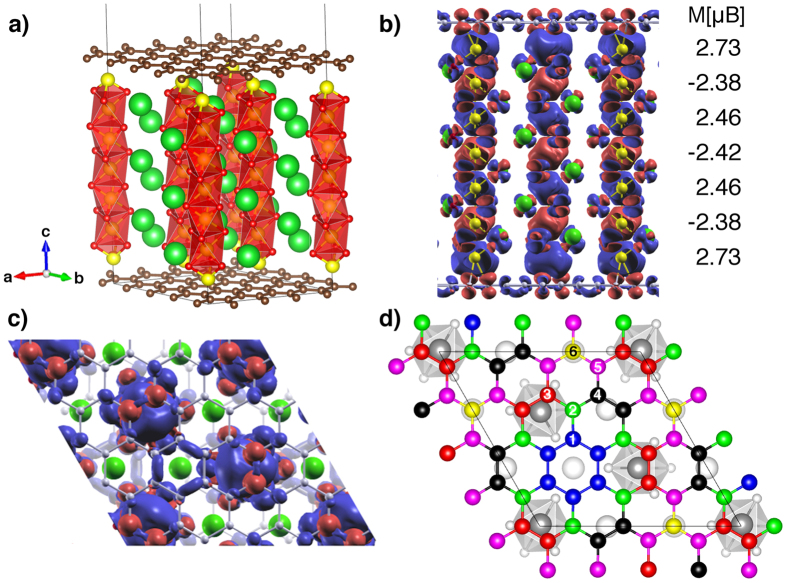
Spin polarization in the graphene–BaMnO_3_ slab. Fully relaxed graphene–BaMnO_3_ structure (**a**) and the corresponding side (**b**) and top (**c**) view of the spin density (*ρ*_↑_ − *ρ*_↓_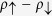). Blue and red correspond to positive and negative isodensities, respectively. The magnetic moment on the Mn atoms are indicated in panel (b). The spin polarization induced on graphene is apparent in panel (c). The six graphene sublattices are numbered and indicated with different colours in panel (d).

**Table 1:** Layer by layer computed magnetic moments (in *μ*_*B*_) on individual atoms in the BaMnO_3_ and graphene-BaMnO_3_ slabs.

	BaMnO_3_			g–BaMnO_3_		
	Mn	O	Ba	Mn	O	Ba
Surface	4.29			2.73		
Surface–1	−2.48	0.118	0.018	−2.38	0.065	−0.024
Surface–2	2.44	0.016	0.039	2.46	0.016	0.021
Surface–3	−2.41	0.000	0.000	−2.42	0.000	0.000

The BaMnO_3_ slab is symmetric. The atomic layers are labelled from the top Mn surface (“Surface”) towards the middle (“Surface–3”) layer.

**Table 2:** Spin polarization and charge transfer in graphene.

Sublattice	1	2	3	4	5	6
M (*μ*_*B*_)	0.009	0.006	−0.034	0.006	0.019	0.011
ΔQ (e^−^)	0.013	0.030	0.024	0.006	−0.009	−0.027

Magnetic moment (M, *μ*_*B*_) and charge transfer (ΔQ, e^−^) per C atom belonging to a given graphene sublattice, as defined in Fig. 3d. Positive (negative) sign of charge transfer indicates charge acquired (donated) by the C atom.
