# Supplementary material for: Graphene-multiferroic interfaces for spintronics applications
Source: Sci Rep. 2016 Aug 23;6:31346. doi: 10.1038/srep31346 (PMC4993994; doi:10.1038/srep31346)
Supplement: Supplementary Information [file srep31346-s1.pdf]

Supplementary Information for:
Graphene-multiferroic interfaces for spintronics applications.

Zeila Zanolli
Forschungszentrum Jülich, PGI-1 and IAS-1, Jülich, D-52425, Germany,
Institute for Theoretical Solid State Physics, RWTH Aachen University, D-52056 Aachen, Germany and
*European Theoretical Spectroscopy Facility (ETSF)**

Convergence tests

Convergence studies have been performed on the energetic ordering of the ferroelectric ($P6_3cm$) and paraelectric ($P6_3/mmc$) phases of bulk $BaMnO_3$. Experiments[1] and first principles calculations performed with hybrid functionals[2] consistently find a ferroelectric ground state for the hexagonal polymorph of $BaMnO_3$. In this work, the correct energetics strongly depends on the real space grid cutoff, making it necessary to use a grid as high as 1200 Ry to be able to distinguish energy differences smaller than 0.5 meV (Figure S1). Furthermore, the k-point sampling needs to be increased up to $6 \times 6 \times 12$ (30-atom cell) to stabilise the result. The role of the functional is also crucial: With the converged parameters and the LDA, the ferroelectric phase is found to be lower than the paraelectric one by 1.2 meV for the 30-atom cell. An overview of these results is reported in Table S1 for structures fully relaxed (atomic positions and lattice vectors) in the $P6_3cm$ and $P6_3/mmc$ symmetry.

All the investigated structures were relaxed until the maximal force on atoms was smaller than 0.001 eV/Å. A supercell approach with more than 30Å of vacuum between the periodic replicae in the [001] direction and a $6 \times 6 \times 1$ sampling of the Brillouin zone were used to model the graphene-$BaMnO_3$ and $BaMnO_3$ slabs.

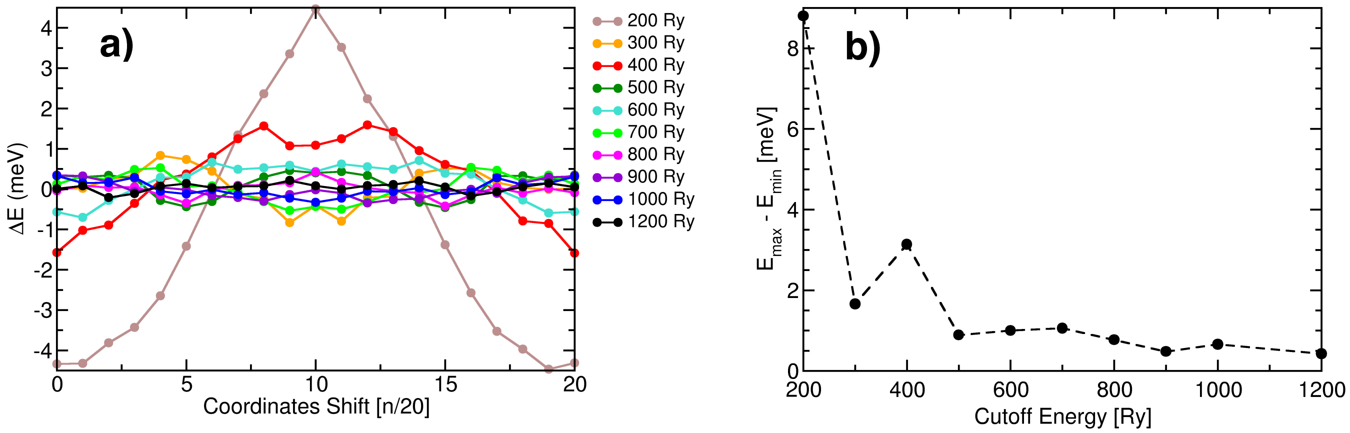

FIG. S1: **The egg box effect:** the total energy changes by displacing the coordinates by a fraction of the unit cell length. The effect can be visualised by plotting the difference between the total energy E and its average $\Delta(E) = E - (E_{max} + E_{min})/2$ for every coordinate shift, at each value of the energy cutoff (a). The accuracy on total energy calculations is given by the width of the egg box oscillation $E_{max} - E_{min}$ for a given cutoff (panel b).

		$\Delta(E)$ [meV]	
cutoff energy (Ry)	k-mesh	**LDA**	GGA
400	$4 \times 4 \times 8$	-2.727	-2.59
1200	$4 \times 4 \times 8$	0.917	0.109
**1200**	$\mathbf{6 \times 6 \times 12}$	**1.176**	-0.070
1200	$8 \times 8 \times 16$	1.04	0.284

TABLE S1: **The effect of various convergence parameters on the energetic ordering of the ferroelectric ($P6_3cm$) and paraelectric ($P6_3/mmc$) phases of hexagonal $BaMnO_3$.** The total energy difference $\Delta(E) = E[P6_3/mmc] - E[P6_3cm]$ is positive for a ferroelectric ground state. The converged parameters are indicated in bold.

Bulk $BaMnO_3$

The LDA functional and the converged computational parameters reproduce the correct energetic ordering between the paraelectric ($P6_3/mmc$) and ferroelectric ($P6_3cm$) phases of bulk hexagonal $BaMnO_3$ (2H–$BaMnO_3$). The calculations have been performed in the $P6_3cm$ primitive cell, which contains 30 atoms and is three times larger than the primitive $P6_3/mmc$ (10 atoms). The lattice parameters and atomic positions were relaxed until the maximal force on atoms was smaller than 0.001 eV/Å and the stress was smaller than 0.01 GPa. The converged lattice parameters are reported in Table S2, together with the computed macroscopic polarization and magnetic moment on Mn atoms.

Atomic relaxation of 2H–$BaMnO_3$ in the $P6_3cm$ phase was also performed imposing a ∼2% in-plane strain to match the 4×4 relaxed graphene cell (in-plane lattice constant 9.848 Å) in view of building the graphene-$BaMnO_3$ interface. The relaxed out-of plane lattice constant is 4.685 Å. Within the present computational framework, the strained 2H-$BaMnO_3$ is a semiconductor with indirect electronic band gap of 1.55 eV between the L and A points of the Brillouin Zone (Figure S2).

The macroscopic polarization of bulk $BaMnO_3$ has been computed with the Berry phase formalism[3], correctly predicting no polarization for the paraelectric phase ($P6_3/mmc$) and P = 0.267 $\mu C/cm^2$ for the ferroelectric phase ($P6_3cm$). The polarization of strained $BaMnO_3$ is 19.037 $\mu C/cm^2$. These values have also been checked with the Wannier-function method. The discrepancy with the experimental value[4] (1.47 $\mu C/cm^2$) is due to strain and to the underestimation of the lattice constant in LDA.

	$a = b$ [Å]	c [Å]	P [$\mu C/cm^2$]	M [μ_B]
$P6_3cm$	9.6519	4.6978	0.267	2.411
$P6_3/mmc$	9.6519	4.6931	0.000	2.409
strained $P6_3cm$	9.8480	4.6847	19.037	2.431
experiment	9.8467[1]	4.8075[1]	1.47[4]	1.3 – 3

TABLE S2: **Computed lattice constant ($a = b$, c), macroscopic polarization (P), and magnetic moment on Mn atoms (M) for fully relaxed bulk $BaMnO_3$.** The computed results for the $P6_3cm$ phase strained to the 4×4 graphene cell (2% in-plane strain) are also reported. The experimental values from Ref.[1] are obtained for the $P6_3cm$ phase at 80K, while the polarization is measured at room temperature in Ref.[4]. Magnetic moments are from Ref.s[1,5,6].

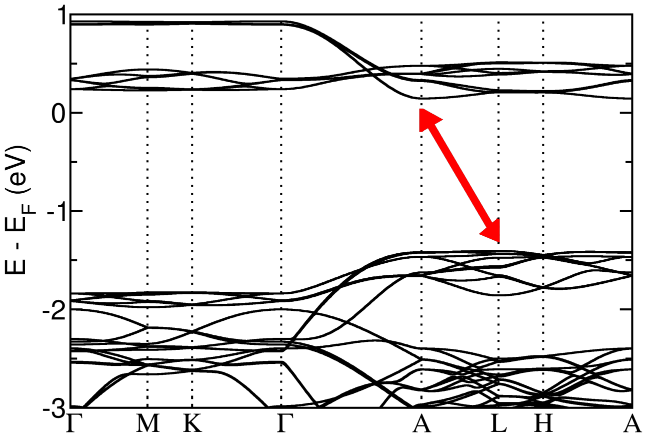

FIG. S2: **Electronic band structure of bulk 2H-$BaMnO_3$ in the ferroelectric phase (space group $P6_3cm$) strained to the 4x4 in-plane graphene lattice constant.** The indirect gap (1.55 eV) between the L and A points of the Brillouin Zone is indicated by an arrow.

The $BaMO_3$ slab and the polar catastrophe

In order to model the graphene–$BaMnO_3$ interface, one can cut a stoichiometric slab along the (0001) plane out of bulk $BaMnO_3$ which is terminated on one side by Mn and, on the other, by BaO_3 atoms. However, the resulting slab is polar and will artificially become metallic (*polar catastrophe*) due to excess electronic charge on either side of

the slab: Each Mn atom has a +4 and each BaO_3 has a −4 electron charge. To avoid this computational artefact, symmetric slabs have been considered, which our calculations correctly predict to be semiconducting (see main text). However, after atomic relaxation, these slabs return to a centrosymmetric structure, losing any initial ferroelectric polarization. For this reason, the present work focuses on the electronic and magnetic interaction between graphene and $BaMnO_3$.

To avoid spurious interaction between the two opposite surfaces it is necessary to model a slab consisting of 13 atomic layers, i.e. 3 times the $BaMnO_3$ unit cell plus a Mn layer.

Graphene on the Mn-terminated $BaMnO_3$ slab: LDA+U

In Mn-based compounds the strong correlation of Mn $3d$ electrons limits the validity of the LDA and GGA approximations to the exchange-correlation functional. These approximations induce a spurious electrostatic self-interaction of the electron charge that overestimates the delocalization of Mn d states[7]. To test how this affects the results presented in this work, we computed magnetization and charge transfer for the graphene-$BaMnO_3$ ground state using the LDA+U approach. We have used the U value obtained by Hong et al[8] by fitting $BaMnO_3$ total energies to Heyd-Scuseria-Ernzerhof (HSE) calculations, i.e. U = 2.7 eV. These calculations show that the inclusion of U does not bring qualitative changes with respect to the LDA results, justifying the use of the LDA approach. The magnetization on Mn atoms is slightly increased with respect to the LDA case (Table S3). The largest change concerns the Mn at the interface with graphene and amounts at about 0.8 μ_B. Most important, charge and magnetization transfer to graphene follow the same trend as in LDA. The difference in the computed magnetization and charge transfer on C atoms is at most 0.015 μ_B and 0.008 e^-, respectively (Table S4).

g–$BaMnO_3$	LDAU			LDA		
	Mn	O	Ba	Mn	O	Ba
Surface	3.55			2.73		
Surface–1	−2.76	0.063	−0.029	−2.38	0.065	−0.024
Surface–2	2.79	0.084	0.028	2.46	0.016	0.021
Surface–3	−2.77	−0.002	−0.001	−2.42	0.000	0.000

TABLE S3: **Layer by layer computed magnetic moments (in μ_B) on individual atoms in the graphene-$BaMnO_3$ slab with and without the U correction.** The $BaMnO_3$ slab is symmetric. The atomic layers are labelled from the top Mn surface ("Surface") towards the middle ("Surface–3") layer.

Sublattice	1	2	3	4	5	6
M (μ_B) LDA	0.009	0.006	−0.034	0.006	0.019	0.011
M (μ_B) LDAU	0.013	0.000	−0.049	0.014	0.028	0.003
ΔQ (e^-) LDA	0.013	0.030	0.024	0.006	−0.009	−0.027
ΔQ (e^-) LDAU	0.009	0.032	0.027	0.004	−0.010	−0.031

TABLE S4: **Magnetic moment (M, μ_B) and charge transfer (ΔQ, e^-) per C atom belonging to a given graphene sublattice computed within the LDA and LDAU formalism**. Positive (negative) sign of charge transfer indicates charge acquired (donated) by the C atom.

Graphene on the Mn-terminated $BaMnO_3$ slab

A detailed analysis of the electronic band structure of the graphene-$BaMnO_3$ (g-BMO) slab has been performed by projecting the band structure (*fat bands*) over the individual orbitals of each atoms. This analysis reveals that the $5s$ electrons of Ba, the $4s$ of Mn and the $2s$ of O do not contribute to the electronic properties of g-BMO in the energy window [-1.5 eV, 0.5 eV] around the Fermi energy. The only relevant states are the $2s$ and $2p$ of C, the Mn $3d$ and the

O 2p. More specifically, the [-1.25 eV, 0.25 eV] region is almost exclusively contributed by the interface atoms, that is graphene and the atoms of the surface $BaMnO_3$ layer. The fat bands analysis including only the interface atoms of the g-BMO slab is illustrated in Fig. S3.

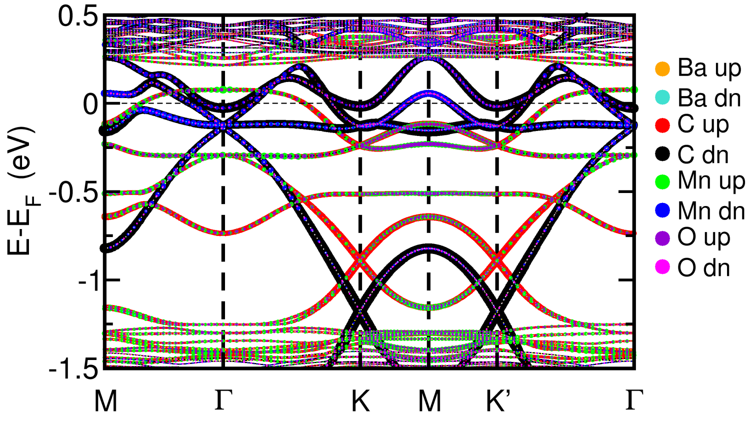

FIG. S3: **Electronic band structure projected on the atomic orbitals (fat bands) for the graphene-$BaMnO_3$ ground state structure: contribution of surface atoms only.** The orbital projection highlights that the energetic levels in the vicinity of E_F are exclusively due to the atoms at the interface between graphene and $BaMnO_3$. The Fermi energy of the hybrid system is taken as reference energy.

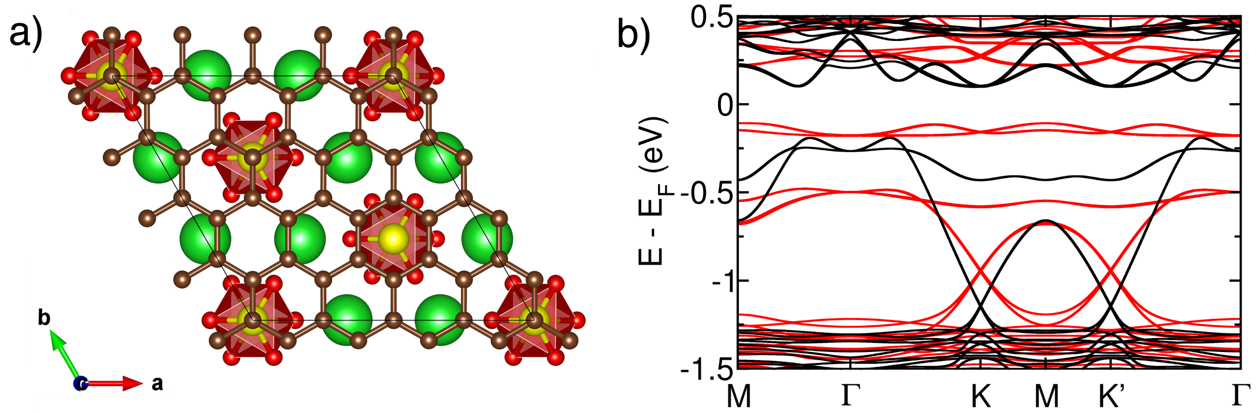

FIG. S4: **Ball-and-stick model (a) and electronic band structure (b) of a metastable phase of the symmetric graphene-$BaMnO_3$ slab.** The graphene/$BaMnO_3$ distance is ∼1.834 Å which allows for a strong C-Mn interaction. The energy difference between this configuration and the ground state is 0.237 eV per unit cell. This metastable structure is a magnetic semiconductor, with an energy gap of 324 meV and 205 meV for majority (red lines) and minority (black lines) carriers. The Dirac cone splitting amounts to 200 meV. The Fermi energy is taken as reference.

Graphene on the BaO_3-terminated $BaMnO_3$ slab

A symmetric $BaMnO_3$ slab terminated with the BaO_3 surface has also been studied and found to be semiconducting. However, in this case, there is no spin polarization at the surface since all the Mn bonds are saturated by O atoms. Next, a 4×4 graphene supercell was placed on both sides of such a slab, as illustrated in Fig. S5.a. After atomic relaxation, the average distance between graphene and the BaO_3 layer is 2.82 Å. The binding energy is -106 meV per C, indicating a weaker bonding with respect to the Mn-terminated surface (binding energy -274 meV per C). The magnetic moment at the surface of the BaO_3-terminated slab are quite small, and the magnetization induced on C atoms is negligible. The electronic band structure of the relaxed slab is reported in Fig. S5.b.

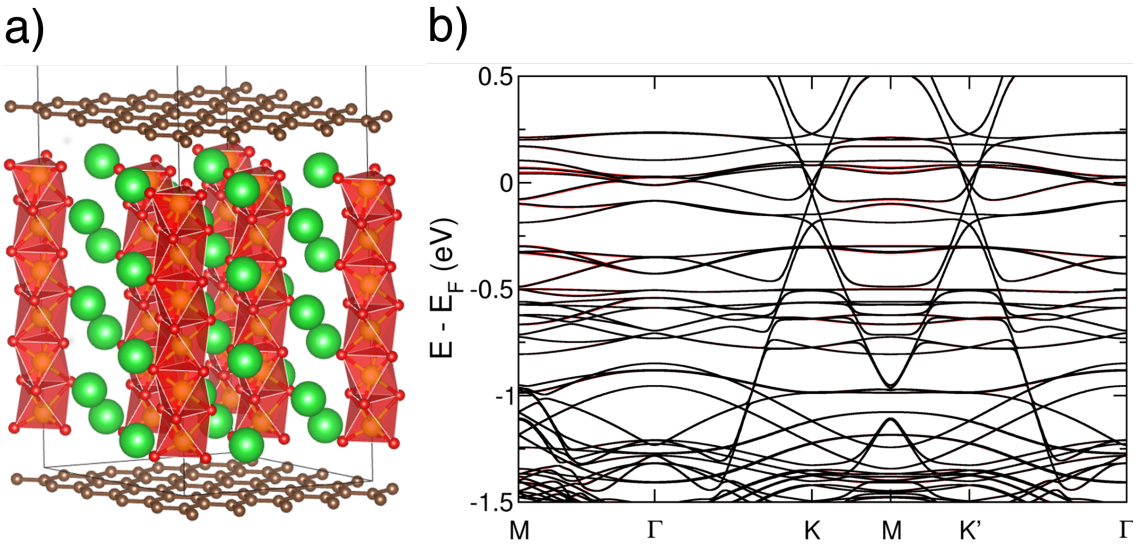

FIG. S5: **Graphene on the BaO_3-terminated $BaMnO_3$ slab.** (a) Relaxed structure and (b) electronic band structure of the graphene-$BaMnO_3$ slab constructed from the BaO_3-terminated surface of $BaMnO_3$. The Fermi energy of the system is taken as reference energy.

---

* Electronic address: `zeilazanolli@gmail.com`

[1] E. J. Cussen and P. D. Battle, Chemistry of Materials **12**, 831 (2000).
[2] J. Varignon and P. Ghosez, Phys. Rev. B **87** (2013).
[3] R. D. King-Smith and D. Vanderbilt, Phys. Rev. B **47**, 1651 (1993).
[4] S. Satapathy, M. K. Singh, P. Pandit, and P. K. Gupta, Applied Physics Letters **100**, 042904 (2012).
[5] A. N. Christensen and G. Ollivier, Journal of Solid State Chemistry **4**, 131 (1972).
[6] J. J. Adkin and M. A. Hayward, Journal of Solid State Chemistry **179**, 70 (2006).
[7] S. Kümmel and L. Kronik, Rev. Mod. Phys. **80**, 3 (2008).
[8] J. Hong, A. Stroppa, J. Íñiguez, S. Picozzi, and D. Vanderbilt, Phys. Rev. B **85**, 054417 (2012).
